# Inhomogeneous magnetization transfer (ihMT) imaging reveals variable recovery profiles of active MS lesions according to size and localization

**DOI:** 10.1162/imag_a_00235

**Published:** 2024-07-24

**Authors:** Lucas Soustelle, Samira Mchinda, Andreea Hertanu, Soraya Gherib, Lauriane Pini, Maxime Guye, Jean-Philippe Ranjeva, Gopal Varma, David C. Alsop, Jean Pelletier, Olivier M. Girard, Guillaume Duhamel

**Affiliations:** Aix Marseille Univ, CNRS, CRMBM, Marseille, France; APHM, Hôpital Universitaire Timone, CEMEREM, Marseille, France; Division of MR Research, Radiology, Beth Israel Deaconess Medical Center, Harvard Medical School, Boston, MA, United States; APHM, Hôpital Universitaire Timone, Service de neurologie, Marseille, France

**Keywords:** myelin MR imaging, remyelination mechanisms, inhomogeneous magnetization transfer, ihMT, multiple sclerosis, MS lesions

## Abstract

This work aims at exploiting the unique myelin specificity of the inhomogeneous magnetization transfer (ihMT) technique to characterize the recovery dynamics of active multiple sclerosis (MS) lesions. IhMT and three other myelin-sensitive techniques, conventional MT, T_1_-weighted, and diffusion tensor imaging, were applied in a 12-month longitudinal study performed on relapsing-remitting MS patients. An exponential recovery model was used to fit the variations over time of the metrics derived from each MR technique within new active lesions. A principal component analysis was performed on the model parameters obtained for all MR myelin-sensitive techniques across all active lesions of all patients to identify specific recovery profiles. The results show that the recovery profiles of myelin-sensitive MR metrics in active MS lesions vary according to the localization and size of lesions. The distance of lesions from the ventricles is positively associated with the recovery rates of ihMTR and T_1w_-MPRAGE: the further the lesion is from the ventricles, the higher the recovery rate of these metrics. Lesion size is positively associated with initial loss and negatively associated with final recovery of ihMTR and other MR metrics: small lesions have lower initial loss and greater final recovery of MR metrics than large lesions. Thanks to the specificity of the ihMT technique for myelin, these features can be interpreted in terms of remyelination. This study thus provides longitudinal in vivo support for the pathological observations of higher remyelination in small lesions compared with large ones and faster remyelination in lesions away from the ventricles. These results support the use of ihMT and other measures for quantifying remyelination rates in clinical studies of remyelination therapies.

## 1 Introduction

Axonal pathology in multiple sclerosis (MS) occurs in focal inflammatory and demyelinating white matter (WM) lesions and within the so-called normal-appearing white matter (NAWM). Some axonal damage is mild and reversible and the mechanisms allowing for the recovery of tissue integrity include remyelination and axonal repair (Franklin et al., 2012;Oh et al., 2019). Remyelination is a spontaneous regenerative process that can occur with high efficiency early in the disease in new MS lesions located in previously unaffected white matter. Once the processes of oligodendrocytes and myelin destruction have ceased completely, usually within few weeks in many new lesions, the reappearance of oligodendrocytes frequently triggers remyelination, giving rise to remyelinated plaques, the so-called shadow plaques (Lassmann et al., 1997;Patrikios et al., 2006;Prineas et al., 1993). Remyelination represents a powerful means of preventing axonal damage attributable to loss of myelin trophic support and hence enables tissue protection.

Surrogate markers for remyelination are required to demonstrate that experimental therapeutic agents promoting remyelination (Franklin et al., 2012) achieve their full effect. Unfortunately, there is no gold standard imaging biomarker for remyelinated lesions nor clinical tools to assess the remyelination status of individual patients (Wang et al., 2019). Different myelin-sensitive MRI methods have been proposed to correlate the changes of their measures (including myelin water fraction (MWF), T_1_, magnetization transfer ratio (MTR), and radial diffusivity (RD)) with the variations of myelin content (Chen et al., 2008;Galbusera et al., 2022;Kitzler et al., 2022;Kolb et al., 2021), but their application in clinical trials led to variable results (Wang et al., 2019) and many remyelination processes of active MS lesions have yet to be characterized*in vivo*.

Inhomogeneous magnetization transfer (ihMT) (Varma et al., 2015) is a refinement of the MT technique that provides unique contrast between tissues relative to MT by isolating dipolar order effects within motion-restricted molecules (Varma et al., 2015) that are weighted by the corresponding dipolar order relaxation time, T_1D_. Because T_1D_is longer in myelinated tissues than any other surrounding tissue in the brain, ihMT images are highly sensitive and specific to myelin. In particular, the specificity of the ihMT signal is much higher than for metrics derived from other myelin-sensitive techniques including MTR and T_1_(Duhamel et al., 2019;Hertanu et al., 2022). IhMT also demonstrated better sensitivity to the MS pathology and a higher correlation with EDSS compared with conventional MT (Rasoanandrianina et al., 2020;Van Obberghen et al., 2018;Zhang et al., 2020).

In this work, the unique specificity of ihMT for myelin was exploited in a longitudinal study performed on relapsing-remitting MS (RRMS) patients to characterize the individual evolution dynamics of new active lesions over a 12-month period. For comparison, three other myelin-sensitive techniques, conventional MT, diffusion tensor (DTI), and T_1_-weighted (T_1w_) imaging were included in the protocol. A time-recovery exponential model was used to fit the variations of ihMT-, MT-, DTI-, and T_1w_-derived metrics measured in new active lesions over time. The parameters of the model included the lesions’ initial values, final values after recovery, and recovery rates. A principal component analysis (PCA) with the ID of the patient from which the lesion(s) came, the size of the lesions, and their localization relative to the ventricles defined as categorical variables, was performed on the model parameters obtained for all MR techniques across all active lesions of all patients with the aim of discovering patterns in recovery. These variables were chosen based on studies that have highlighted the importance of size (Franklin & ffrench-Constant, 2008) and localization in relation to the ventricles (Goldschmidt et al., 2009;Patrikios et al., 2006) in the ability of a lesion to remyelinate.

## 2 Methods

### 2.1 Population and clinical assessment

Nine patients with RRMS (8:1 female:male; mean age = 32.8 years, range = 21-51 years, mean disease duration = 66.6 ± 73.6 months and median Expanded Disability Status Scale (EDSS) score = 1.0, range = 0-3.5) were enrolled in a 12-month longitudinal MR study. Inclusion was based on the occurrence of at least one active lesion on a contrast-enhanced T_1_-weighted brain scan performed less than 15 days earlier. Patients were scanned every other month during 6 months and a final scan was conducted in the 12th month. Scanning time points are hereafter referred to as M_0_, M_2_, M_4_, M_6_, and M_12_. Patients’ characteristics and clinical data at M_0_are presented inTable 1. Eight control subjects (4:4 women:men; mean age = 29.4 years, range = 21-39 years) were scanned twice (at M_0_and M_12_) to provide a control database for comparison. Patients and control subjects provided informed consent to participate in this research study, which received the approval of the local research ethics committee (CPP Sud Méditerranée 1).

**Table 1.** Subject demographics.

					Lesions			
Patient #	Gender - age (M _0_ )	Disease duration (M _0_ )	EDSS M _0_ /M _12_	Treatment during follow-up		Month of detection	Core size (mm ^3^ )	Localization w/r ventricles *
1	F – 27 y	61 m	1.5/1	None (M _0_ ), natalizumab (M _2_ -M _12_ )	L1	M _0_	118	Pro.
					L2	M _0_	589	Dis.
					L3	M _0_	1095	Pro.
					L4	M _0_	131	Dis.
					L5	M _0_	152	Dis.
					L6	M _0_	899	Pro.
					L7	M _0_	276	Dis.
					**L8**	M _2_	239	Dis.
					**L9**	M _2_	226	Pro.
					L10	M _2_	617	Pro.
					L11	M _2_	238	Pro.
2	M – 42 y	214 m	1/1	None	**L12**	M _0_	1340	Pro.
					**L13**	M _0_	648	Dis.
3	F – 21 y	59 m	3.5/2.5	None (M _0_ ), natalizumab (M _2_ -M _4_ ), rituximab (M _6_ -M _12_ ),	L14	M _0_	100	Pro.
					**L15**	M _0_	62	Pro.
					L16	M _0_	371	Dis.
					**L17**	M _0_	218	Dis.
					**L18**	M _0_	74	Dis.
					L19	M _0_	143	Pro.
					L20	M _0_	83	Dis.
					L21	M _0_	32	Pro.
					L22	M _0_	112	Pro.
					L23	M _0_	83	Dis.
					L24	M _0_	78	Dis.
					**L25**	M _2_	30	Pro.
4	F – 42 y	58 m	1.5/1	None (M _0_ )Fingolimod (M _6_ -M _12_ )	L26	M _0_	110	Pro.
					L27	M _0_	40	Dis.
					L28	M _0_	108	Dis.
					L29	M _0_	47	Pro.
					**L30**	M _0_	72	Pro.
5	F – 23 y	4 m	0/0	None	**L31**	M _2_	57	Dis.
6	F – 51 y	160 m	0/0	None	L32	M _0_	375	Dis.
					L33	M _0_	635	Dis.
					L34	M _0_	288	Dis.
					**L35**	M _0_	547	Dis.
					L36	M _0_	312	Dis.
					L37	M _0_	27	Dis.
					**L38**	M _0_	12	Dis.
					**L39**	M _0_	157	Dis.
					L40	M _0_	72	Dis.
					L41	M _2_	63	Dis.
					**L42**	M _2_	43	Dis.
7	F – 28 y	6 m	0/1	None	L43	M _0_	45	Dis.
					L44	M _0_	50	Pro.
					**L45**	M _0_	41	Dis.
					**L46**	M _0_	22	Dis.
					L47	M _2_	110	Pro.
8	F – 39 y	34 m	1.5/1	Fingolimod Rituximab (M _2_ -M _12_ )	L48	M _0_	682	Pro.
9	F – 22 y	3 m	0/0	None (M _0_ )Dimethyl fumarate (M _6_ -M _12_ )	L49	M _0_	124	Pro.
					L50	M _0_	357	Pro.
					L51	M _0_	14	Dis.
					L52	M _0_	66	Dis.

* Pro.: lesions proximal to ventricles (<10 mm). Dis.: lesions distal from ventricles (>10 mm). Lesions indicated in bold correspond to those for which the recovery model failed to fit the signal dynamics with R2adj < 0.6 for ihMTR (L8, L9, L15, L17, L18, L20, L25, L31, L42, and L46), MTR (L8, L25, L31, L35, L42, and L46), RD (L8, L12, L13, L25, L30, L31, L35, L38, L39, L42, L45, and L46), and MPRAGE (L42).

### 2.2 MRI protocol

The longitudinal MR study was performed on a 1.5T MRI system (MAGNETOM Avanto, Siemens Healthineers, Erlangen, Germany) with a body coil for transmission and a 32-channel receive-only head coil. The MR protocol included 1-mm isotropic magnetization prepared rapid acquisition of gradient echo (MPRAGE; T_1_-weighted), and fluid-attenuated inversion recovery (FLAIR; T_2_-weighted) that were used for image registration and lesion segmentation. The T_1_-weighted MPRAGE was also considered for myelin imaging along with a sensitivity-enhanced 3D ihMT gradient echo (GRE) sequence (Mchinda et al., 2018), a conventional 3D MT-GRE sequence and a multislice 2D Diffusion-Weighted Spin-Echo Echo-Planar Imaging sequence, which were acquired with sequence parameters provided inTable 2. Additionally, at M_0_, at the end of the protocol, spin-echo T_1w_images were acquired pre- and postintravenous injection of gadoterate meglumine (2 mL/kg, concentration 0.5 mmol/mL, Dotarem, Guerbet, Roissy CdG, Cedex, France) for active lesion detection by contrast-enhancement.

**Table 2.** Sequence parameters used for ihMT, MT, and DTI imaging.

		3D ihMT-GRE	3D MT-GRE	2D DTI-EPI
Contrast parameters		Sensitivity-enhanced ihMT preparation * : 0.5 ms Hann-shaped pulse Pulse repetition time Δt= 1.0 ms Number of pulses per burst of saturation N _pulses_ = 12 Saturation power B _1,RMS_ = 5.5 µT Frequency offset, ∆f = 8 kHz	7.68-ms gaussian pulse 1.5 kHz frequency offset Applied flip angle of 500°	1 reference (b = 0 s/mm²) 64 directions (b = 800 s/mm²)AP/PA phase encoding
Readout parameters	Matrix	128 x 100 x 80	128 x 100 x 80	104 x 104 x 72
	Voxel size	2.0 mm iso.	2.0 mm iso.	2.5 x 2.3 x 2.3 mm ^3^
	timing	TR/T _RO_ /TE = 67.9/6.2/3.0 ms9 readout segment per preparation	TR/TE = 16.0/3.0 ms 1 readout segment per TR	TR/TE = 3855/68.2 ms
	Readout flip angle	7°	10°	180/90°
	Receiver bandwidth	370 Hz/voxel	370 Hz/voxel	1658 Hz/voxel
Acquisition time		9’06”	2’40”	9’39”

* As reported inMchinda et al. (2018).

### 2.3 Parametric images

The conventional MT ratio (MTR) was calculated as:

$$MTR=1-\frac{MT}{MT_{0}}$$, (1)

where MT and MT_0_correspond to the saturated and nonsaturated rigidly coregistered 3D MT-GRE images (corrected for Gibbs-ringing artifacts with an isotropic 3D-cosine kernel), respectively.

The ihMT ratio (ihMTR) was computed from the four MT-weighted and reference images derived from the 3D ihMT-GRE sequence as follows (Mchinda et al., 2018;Varma et al., 2015):

$$ihMTR=\frac{MT^{+}\text{​}+MT^{-}-MT^{\pm }-MT^{\mp }}{MT_{0}}$$, (2)

where$MT^{+}$and$MT^{-}$correspond to MT-weighted images obtained with saturation at single frequency offsets +∆f and -∆f, respectively;$MT^{\pm }$and$MT^{\mp }$correspond to MT-weighted images obtained with dual-frequency offset saturation by alternating the frequency of the RF saturation pulses from +∆f to -∆f every successive pulse;$MT_{0}$corresponds to the nonsaturated reference image. The MT-weighted magnitude images were denoised using the MP-PCA (Grussu et al., 2020;Veraart et al., 2016) routine from the MRtrix3 package (v. RC301) (Tournier et al., 2019), corrected for Gibbs-ringing artifacts with an isotropic 3D-cosine kernel, and combined to generate ihMTR maps based onequation 2following application of a dedicated motion correction algorithm (Soustelle et al., 2020). A bash-based wrapper comprising the aforementioned steps is available at:https://github.com/lsoustelle/ihmt_proc(hash #c9bb409).

Radial diffusivity (RD) was computed from diffusion-weighted images using the DESIGNER pipeline (Ades-Aron et al., 2018) based on the MRtrix3 library. The preprocessing steps included MP-PCA denoising (Veraart et al., 2016), Gibbs artifacts removal (Kellner et al., 2016) followed by motion correction and distortion correction steps (Andersson et al., 2003,^2016^), prior to tensor estimation.

T_1w_-MPRAGE images were preprocessed by a bias-field correction using Nick’s N3 algorithm (Tustison et al., 2010) implemented in the Advanced Normalization Tools (ANTS; v. 2.3.3) (Avants et al., 2009), and after atlas-based brain extraction using the*antsBrainExtraction.sh*routine (Avants et al., 2010) from the MNI152 atlas (symmetric ICBM 2009a).

### 2.4 Image postprocessing

The postprocessing procedures described hereafter were designed to allow quantitative measurements and comparison of MR metrics in masks of active lesions, masks of NAWM, and masks of normal white matter (NWM) at different time points of the longitudinal study.

First, for each subject, T_1w_-MPRAGE images, FLAIR images, ihMTR maps, MTR maps, and RD maps acquired at all time points were rigidly registered onto the anatomic T_1w_-MPRAGE volume acquired at M_0_using ANTS. Masks of active lesions, nonactive lesions, and contralateral NAWM were then derived as described in Sections 2.4.1 to 2.4.3. Masks of NWM in brain areas corresponding to the areas of MS lesions were derived from the control groups as described inSection 2.4.4.

#### 2.4.1 Masks of active lesions

**Fig. 1.**
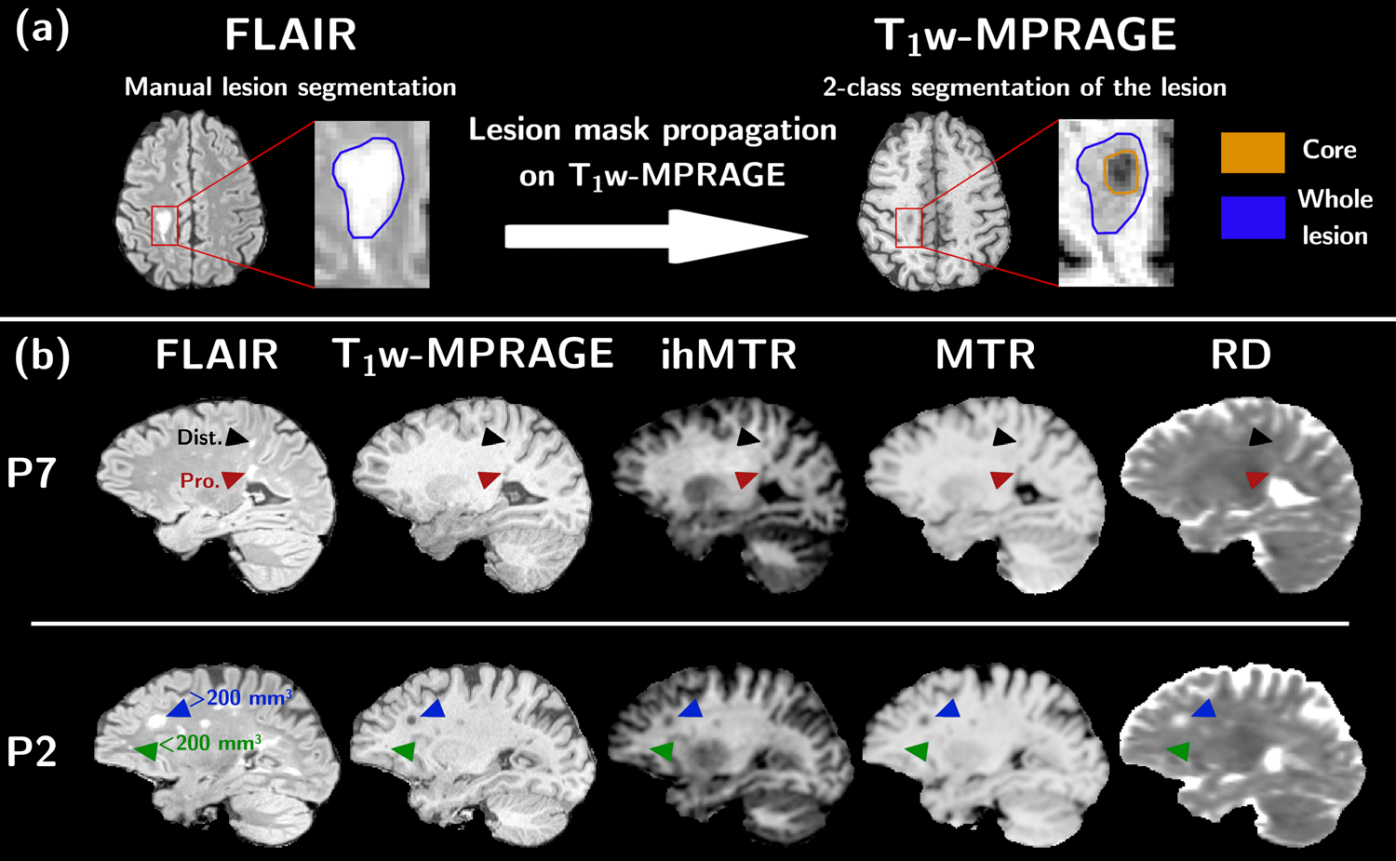
Sketch of the lesion core submask creation (a). The mask of the lesion, manually drawn on the FLAIR image, is propagated on the T_1W_-MPRAGE image. A two-class segmentation performed with the Atropos’ k-means clustering algorithm applied on the T_1W_-MPRAGE image creates the lesion core submask. Representative sagittal views of FLAIR, T_1w_-MPRAGE, ihMTR, MTR, and RD images for two different patients (b) with examples of lesions proximal (Pro., red arrows) and distal (Dist., black arrows) from the ventricles (patient P7), as well as large (> 200 mm^3^, blue arrows) and small (< 200 mm^3^, green arrows) lesions (patient P2).

Lesions following the MS diagnosis criteria (Filippi et al., 2016) and showing T_1_contrast-enhancement postgadolinium injection at M_0_, as well as new lesions detected at M_2_were categorized as active MS lesions. They were manually segmented and labeled by an expert (S.G.) on the FLAIR images. Lesions whose long-axis size was inferior to 3 mm were discarded to limit potential registration-based misalignment along time. A total of 52 lesions met the criteria and were analyzed. Each of the 52 lesion masks was subsequently subdivided into two classes using Atropos’ k-means clustering algorithm (Avants et al., 2011) and based on the initial T_1w_-MPRAGE signal intensity (Thaler et al., 2015). The class with the lowest relative T_1w_intensity was defined as the*core*of the lesion (Fig. 1a), while the second class defined the*edge*of the lesion. The resulting subsegmented masks were then propagated at all time points. All analyses reported in this study were performed in the core of the lesions only (the distribution of ratios between the core volume and the entire volume of active lesions is provided in Supplementary Material,Fig. S1).

##### 2.4.1.1 Active lesions labeling

The 52 active lesions were labeled according to three categorical criteria including their patient ID, their size, and their localization relative to the ventricles ( Table 1 ).

(i) *Patient ID*: little evidence supports that the extent of myelin regeneration in response to a demyelinating insult is patient dependent, with regeneration levels demonstrating high intrapatient variability (Bodini et al., 2016). In an attempt to identify potential patient-specific profiles of remyelination in the dynamics of MR metrics during lesions recovery, all the lesions of a given patient were labeled with the same patient ID.
(ii) *Size*: In the remyelination process, oligodendrocyte precursor cells (OPC) need to be recruited to lesions from surrounding intact tissue. Lesion size affects remyelination efficiency since larger lesions require a greater impetus for OPC recruitment than smaller ones (Franklin & ffrench-Constant, 2008). In an attempt to identify a lesion size effect in the pattern of remyelination, the 52 active lesions of the dataset were classified into 2 categories: Lesions with a core size less than 200 mm^3^were labeled*small,*and lesions with a core size larger than 200 mm^3^were labeled*large*(Fig. 1b). The threshold value of 200 mm^3^was determined based on the observation of the lesion size histogram for which we identified 2 groups: 33 lesions (of the 52) lay in the restricted interval [12 mm^3^-157 mm^3^] and 19 lesions (of the 52) in the wide interval [218 mm^3^-1340 mm^3^].
(iii) *Localization*: Recent studies highlighted the importance of the lesion localization. In particular, a distance close to the lateral ventricles was associated with a lower extent of remyelination (Goldschmidt et al., 2009). A periventricular gradient of structural alterations and inflammation, characterized by lower MTR values and greater activation of innate immune cells in MS lesions located within 7-10 mm of the ventricular CSF compared with lesions further away, was also reported (Liu et al., 2015;Poirion et al., 2021). In an attempt to identify a localization effect in the pattern of remyelination, the 52 active lesions were classified into 2 categories based on their distance to the ventricles. The closest 3D Euclidian distance between the lesion’s centroid and the lateral ventricles surface was measured manually (using the ruler tool in ITK-SNAP (Yushkevich et al., 2006)). Lesions less than 10 mm from the ventricles were labeled as*proximal*(22/52 lesions), while lesions more than 10 mm were labeled as*distal*(30/52 lesions) (Fig. 1b).

#### 2.4.2 Masks of nonactive lesions

Masks of nonactive WM lesions were created at each time point by manual segmentation on the FLAIR images of lesions not showing Gd contrast enhancement at M_0_. For each subject, the union of the masks from all time points defined the final masks of nonactive lesions. This procedure reduced potential mismatches induced by multiple acquisition aspect (partial volume effects due to field-of-views positioning at each time point) and multiple registrations (interpolation-related smoothing).

#### 2.4.3 Masks of contralateral normal-appearing white matter

To investigate the follow-up behavior of active lesions, contralateral masks located in the NAWM and common at all time points were created and used for comparison. Hence, following the detection of active lesions at M_0_or M_2_, we performed (i) lesion filling of all lesions (Battaglini et al., 2012;Guo et al., 2019) on the T_1w_-MPRAGE image; (ii) multistage rigid, affine, and symmetric diffeomorphic registration (SyN) of the MS patient’s T_1w_-MPRAGE image to the standard MNI152 space (symmetric ICBM 2009a) (Avants et al., 2011); (iii) forward transformation of the active lesion mask into the standard space; (iv) left-right mask flipping; and (v) backward transformation of the flipped mask image into the native subject space. Then, to avoid potential overlap of the contralateral masks with altered WM (e.g., lesion) or non-WM areas (e.g., gray matter or ventricles due to imperfect nonlinear registration), we refined the contralateral masks. For that, a mask of the whole NAWM was created at M_0_by (i) use of FreeSurfer (Dale et al., 1999) to segment WM in the subject space at M_0_and (ii) removal of all active and nonactive lesion masks (segmented at all time points) from the WM segmentation. Then, we calculated the intersection of the contralateral masks with the whole NAWM mask to create refined contralateral masks, which were propagated at all time points and used for analyses.

#### 2.4.4 Masks of normal white matter in areas corresponding to lesions areas

For the purpose of comparison of MR metrics in lesions with normal values, a template-based mask of NWM corresponding rigorously to the areas of lesions was created from images of all control subjects as follows: (i) multistage rigid, affine, and symmetric diffeomorphic registration (SyN) of the subject’s T_1w_-MPRAGE image to the MNI152 space; (ii) application of the estimated transformations on quantitative ihMTR, MTR, and RD maps; (iii) cross-subjects averaging of maps at M_0_and M_12_; and finally (iv) projection of the active lesion masks of MS patients into the created template space, which were refined using the MNI template’s paired WM probability image thresholded at 90%.

### 2.5 Dynamic model of MR signal recovery in remyelinating lesions

For each time point (t_i_), the relative variations (*RV_p_*) of ihMTR, MTR, RD, and T_1w_-MPRAGE signal values were calculated in the core of the 52 active lesions by considering the corresponding reference values measured in contralateral NAWM as follows:

$$RV_{P}\left(t_{i}\right)=\frac{P^{L}\left(t_{i}\right)-P^{NAWM}\left(t_{i}\right)}{P^{NAWM}\left(t_{i}\right)}$$, (3)

where$P^{L}(t_{i})$and$P^{NAWM}(t_{i})$correspond to the mean values of the MR parameter*P*at the given time point*t_i_*in the core of a lesion and in the corresponding contralateral NAWM region, respectively. Note that the choice of analyzing the relative variations of the parameters rather than their absolute values was made to include T_1w_-MPRAGE-derived metrics, which could not be compared from patient to patient due to the lack of a reference signal value and the qualitative nature of these kinds of MR images.

An exponential recovery model was then used to fit the temporal dynamics of*RV_P_*s for each MR technique in their recovery phase:

$$RV_{P}\left(t\right)=\left(RV_{P}\left(t_{0}\right)-RV_{P}\left(\infty \right)\right)\times e^{-R\left(t-t_{0}\right)}+RV_{P}\left(\infty \right)$$, (4)

where$RV_{p}(\infty )$is a free parameter determining the asymptotic recovery value of the MR parameter*P*and*R*a free parameter denoting the recovery rate of the MR parameter*P*from its onset loss ($RV_{p}(t_{0})$) to$RV_{p}(\infty )$.$RV_{p}(t_{0})$was a fixed parameter of the model with the time*t_0_*, empirically determined considering the typical period during which an MS lesion is inflammatory demyelinating and shows Gd enhancement (≤4 weeks (Filippi et al., 2019)) according to the following methodology: for each active lesion detected at M_0_,equation 4was fitted to the temporal dynamics of*RV_p_*s using M_0_for t_0_and the adjusted coefficients of determination (*R²_adj_*) were calculated. A value of 0.6 for*R²_adj_*was chosen as a validity criterion of the model: in the case where*R²_adj_*was higher than 0.6 for a particular MRI contrast, the model parameters for that contrast in that lesion were kept for the subsequent analyses. In the case where*R²_adj_*was lower than 0.6, the fit procedure was performed again using M_2_for*t_0_*(omitting the data from M_0_). If*R²_adj_*resulting from the new fit was higher than 0.6, the model parameters for that contrast in that lesion were kept for the subsequent analyses, otherwise, the model parameters for that contrast in that lesion were discarded. For new lesions detected at M_2_, the fits were performed using M_2_for t_0_and the lesion was kept for the subsequent analyses only if*R²_adj_*> 0.6. Note that for RD, even though it is expected to increase with demyelination at t_0_and recover according to an exponential decay model, the terminology*onset loss*and exponential recovery were kept for easier comparison with the other metrics.

### 2.6 Statistics and principal component analysis (PCA)

Mean values of ihMTR, MTR, and RD were calculated in masks of active lesions’ cores, NAWM and NWM at*t_0_*and M_12_. Differences between groups were tested by first examining the homogeneity of variance using the Bartlett test, and then by performing a one-way ANOVA Welch analysis followed by post hoc Games–Howell tests corrected for multiple comparisons using the Holm–Bonferroni procedure (MATLAB, The MathWorks, Natick, Massachusetts, USA). Corrected p-values below 0.05 were considered significant.

The mean coefficient of variation (CoV) of MR parameters in NWM (from the controls group) between*t_0_*and M_12_allowed estimation of the variability in MR parameters induced by normal physiological processes within the longitudinal study period. The mean CoV was calculated with the individual CoVs, i.e., the ratio between the standard deviation and the mean of the parameter values at t_0_and M_12_, averaged over the regions corresponding to the active lesions. In a similar way, the mean CoV of MR parameters in the contralateral NAWM of patients between t_0_and M_12_allowed estimation of the variability in MR parameters within the study period to act as reference for normalization of any changes attributed to remyelination.

Correlation analyses (Pearson test, correlation coefficient ρ²) were performed to evaluate the association between the RV_p_values of different MR parameters at t_0_and at M_12_. This analysis aims at evaluating the degree of complementarity of these techniques of variable specificity. Intraparameter correlation analyses between RV_p_values at t_0_and at M_12_were also performed to evaluate the predictive capability of the MR metrics*.*A Holm–Bonferroni corrected p-value < 0.05 was considered significant for all correlation analyses. Comparison of correlation coefficients between ihMTR and RD and T_1W_-MPRAGE, and between MTR and RD and T_1W_-MPRAGE were tested using Steiger’s modification of Dunn and Clark’s z approach for overlapping correlations based on dependent groups (Steiger, 1980) implemented in the*cocor*package (Diedenhofen & Musch, 2015) in R (v. 4.3.0).

In order to identify lesions with similar recovery profiles (i.e., lesions that have similar parameter values output from the recovery model applied on the MRI techniques), a PCA was conducted on the 12 active variables (*onset loss, asymptotic recovery,*and*recovery rate*of the 4 MR techniques). The*recovery rate R*was expressed in month^-1^while the*onset loss*(reported as$\left|RV_{p}(t_{0})\right|$) and the*asymptotic recovery*(reported as 1+$RV_{P}(\infty )$for MTR, ihMTR, T_1w_-MPRAGE, and 1-$RV_{P}\left(\infty \right)$for RD) values were expressed as percentages of the parameter*P*in NAWM taken for reference. For example, an ihMTR*onset loss*of 80% means that the value of ihMTR in the lesion at t_0_is equal to 20% of its value in NAWM. Conversely, an asymptotic recovery of 85% means the value of ihMTR in the lesion att(∞)is equal to 85% of its value in NAWM. The lesions’ patient ID, size, and localization relative to the ventricles were defined as additional*categorical variables*. A 2-dimension regularized iterative PCA for imputation of missing data was performed using the missMDA and FactorMineR packages (Josse & Husson, 2016) implemented in R (R Core Team, 2022) to account for discarded lesions.

## 3 Results

### 3.1 Variations of metrics in different tissues

Representative evolution of the different MR contrasts over time in normal appearing tissue and in an active lesion can be appreciated inFigure 2. Quantitative analyses showed that MR parameter values in NWM were not significantly different between t_0_and M_12_(Fig. 3). The CoVs of MR parameters measured in NWM were 0.9% for ihMTR, 0.2% for MTR, and 0.8% for RD. MR parameter values in contralateral NAWM were also not different between t_0_and M_12_, but values in contralateral NAWM were significantly different than those in NWM at each time point (Fig. 3). The CoVs of MR parameters measured in contralateral NAWM were 2.5% for ihMTR, 0.7% for MTR, and 1.4% for RD. Finally, MR parameters values in lesions were significantly different between t_0_and M_12_and significantly different than the values in NWM and contralateral NAWM (Fig. 3).

**Fig. 2.**
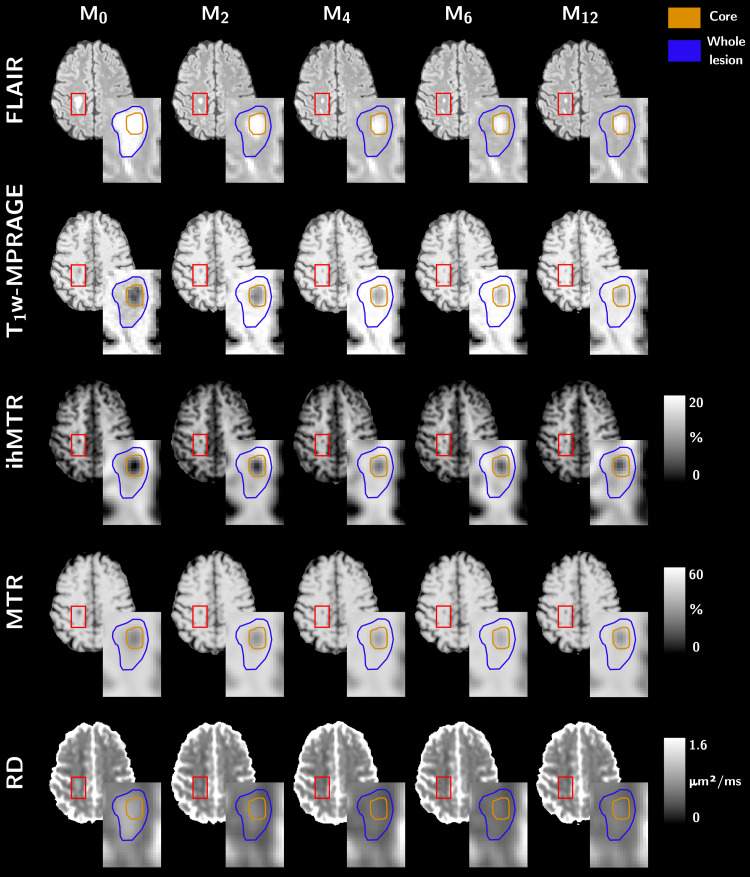
Representative axial images from a patient showing the evolution of the different MR contrasts over time in normal appearing tissue and in an active lesion (zoom inserts). The blue and orange lines delineate the whole lesion and the lesion core, respectively.

**Fig. 3.**
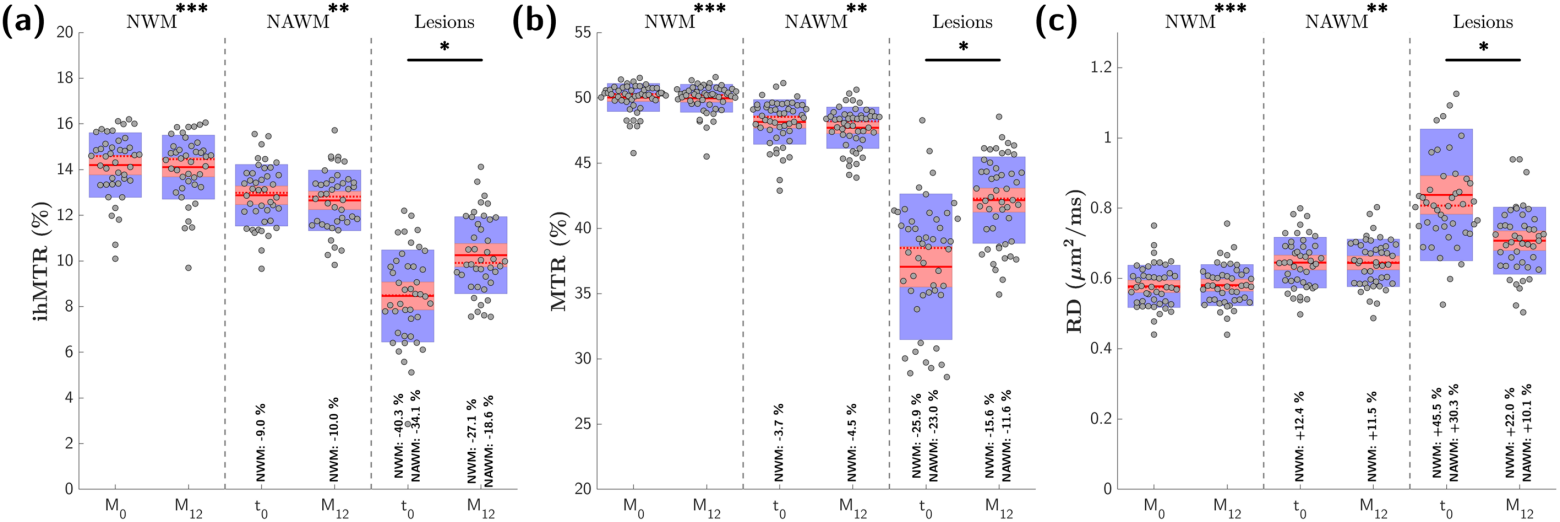
Boxplots of ihMTR (a), MTR (b), and RD (c) average values in NWM, NAWM, and lesions. Solid and dashed red lines indicate mean and median values of the distributions, respectively. Red boxes span from mean to ± standard deviation (SD), and blue boxes span from mean to ± 1.96xSD. Values in NWM at M_0_and M_12_were not different, nor were the values in NAWM at M_0_and M_12_. Conversely, values in lesions were significantly different (p<0.05, corrected for N = 6 comparisons) at M_0_and M_12_(*). Values in NWM (at both M_0_and M_12_) were significantly different than values in NAWM and values in lesions at t_0_and at M_12_(***). Values in NAWM (at both M_0_and M_12_) were significantly different than values in lesions at t_0_and at M_12_(**). Average relative variation of MR parameters evaluated in lesions with respect to NWM and NAWM is reported, as well as that evaluated in NAWM with respect to NWM. Boxplots were rendered using the notBoxPlot package (https://github.com/raacampbell/notBoxPlot) implemented for Matlab (R2017b, The Mathworks Inc., Natick, MA, USA)

### 3.2 Correlation analyses

**Fig. 4.**
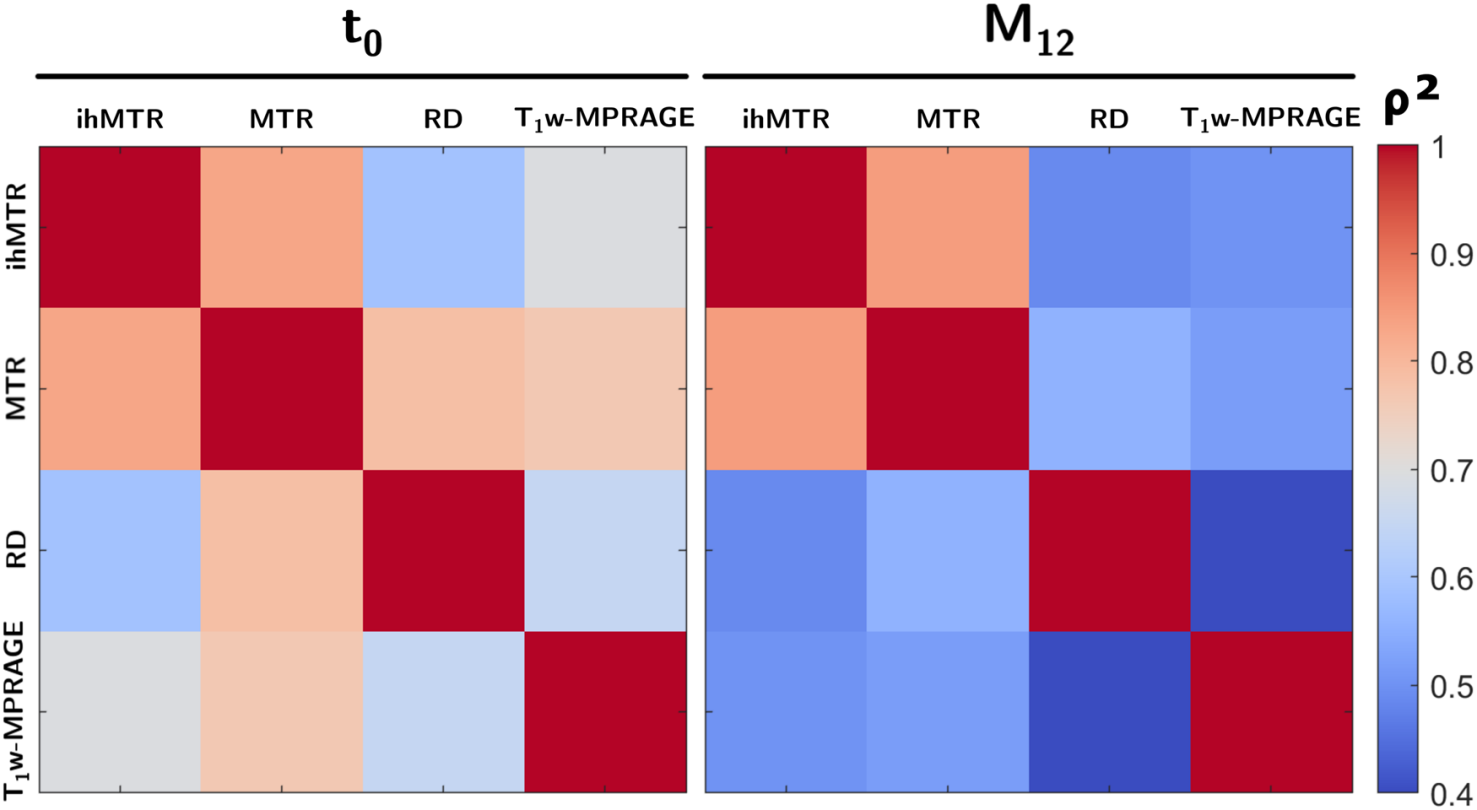
Correlation matrices (Pearson coefficient, ρ^2^) between the RV_p_values (N = 52 active lesions) of MR parameters at t_0_(left) and at M_12_(right).

Interparameter correlation analyses (Fig. 4and Supplementary Material,Fig. S2) between relative variations indicate strong associations between ihMTR and MTR at both t_0_and M_12_(ρ² > 0.83, p < 0.05). On the other hand, at t_0_, ihMTR was significantly less correlated with RD and T_1w_-MPRAGE (ρ² < 0.61, p < 0.05) than was MTR (ρ² > 0.74, p < 0.05).

Intraparameter correlations between t_0_and M_12_(Supplementary Material,Fig. S3) indicate stronger association for ihMTR (ρ² = 0.59, p < 0.001) compared with MTR (ρ² = 0.42, p < 0.001). Correlations for other metrics were low (ρ² = 0.21, p = 0.01 for RD and ρ² = 0.15, p = 0.02 for T_1w_-MPRAGE).

### 3.3 Recovery of MR metrics in active lesions

**Fig. 5.**
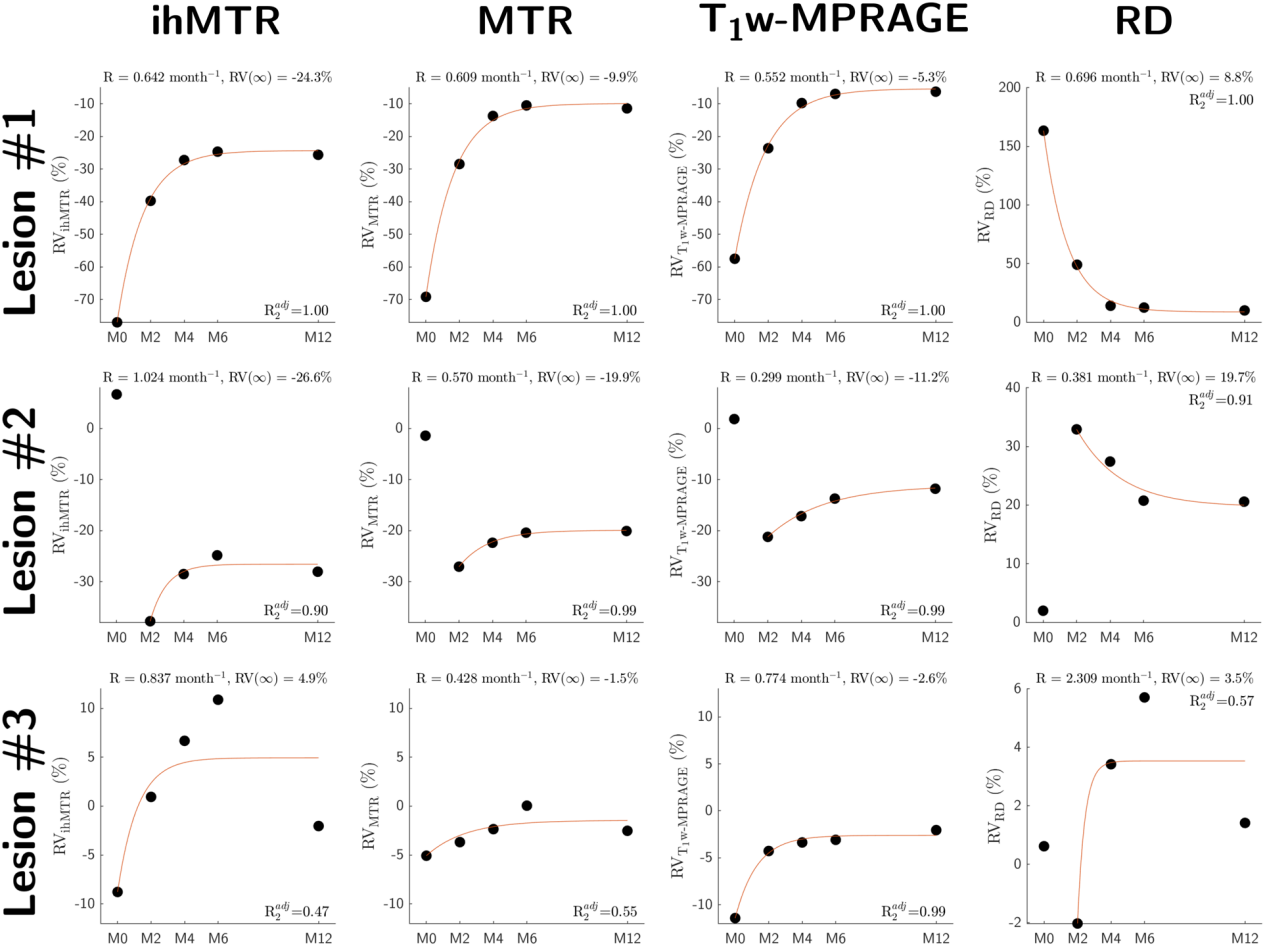
Temporal dynamics of the MR parameters RV values (relative variation between the parameter value in lesion and in NAWM) fitted by the 3-parameter exponential recovery model for 3 different cases: R^2^_adj_> 0.6 and t_0_= M_0_(lesion#1), t_0_= M_2_(lesion#2), and R^2^_adj_<0.6 (lesion#3).

Illustrative temporal dynamics of RV_P_values fitted by the exponential recovery model are illustrated inFigure 5for cases where R²_adj_> 0.6 and t_0_= M_0_(Fig. 5, lesion #1), R²_adj_> 0.6 and t_0_= M_2_(Fig. 5, lesion #2), and R²_adj_< 0.6 (for all MR parameters but T_1w_-MPRAGE,Fig. 5lesion #3). Over the 52 lesions, a total of data from 10 lesions for ihMTR, 6 for MTR, 12 for RD, and 1 for T_1w_-MPRAGE were discarded (Table 1) based on the threshold value of 0.6 for R²_adj_.

### 3.4 Principal component analysis of the dynamic recovery dataset

**Fig. 6.**
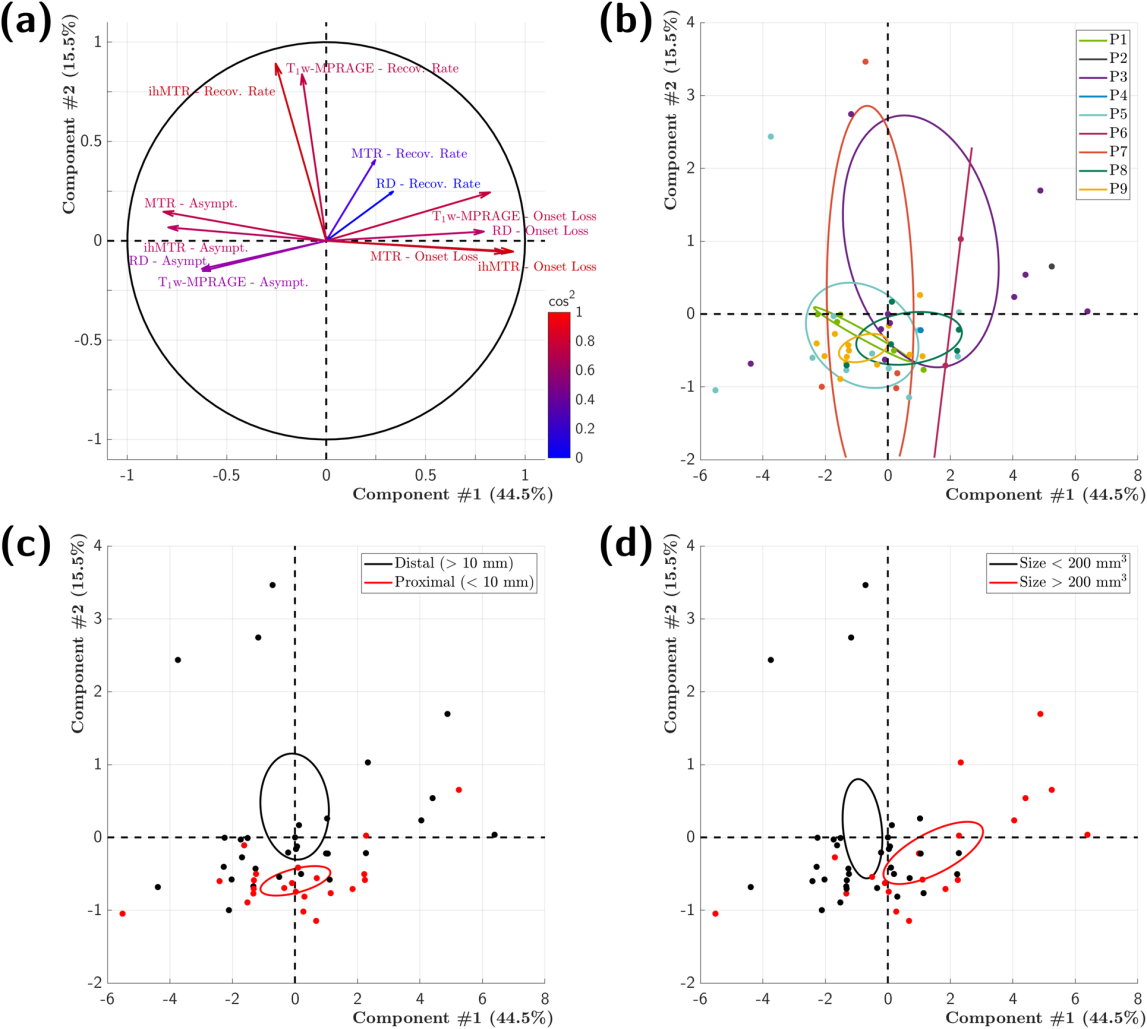
PCA. Loading (correlation values, ρ (Table 3)) plot of the active variables (onset loss, asymptotic recovery, and recovery rate of MR parameters) colored as a function of their quality of representation (cos^2^) on the two first components (a). Score plots of the individuals (lesions) colored as a function of the values of the categorical variables (lesion patient’s ID (b), lesion’s localization (c), and lesion’s size (d)) and 95% confidence ellipses.

The first 2 principal components (PC1 and PC2) of the PCA account for 60% of the total variance of the dataset, and the first 3 components account for more than 70%. The*onset loss*and the*asymptotic recovery*of all MR metrics are the active variables loading on PC1 (Fig. 6a, loadings plot), although the correlations of RD and T_1w_-MPRAGE*asymptotic recovery*variables are lower (ρ < 0.7,Table 3).*Onset loss*variables load oppositely to the*asymptotic recovery*variables on PC1, thus indicating negative correlation between these variables: the higher the initial loss of MR metrics values in active lesions, the lower their final recovery values, and vice versa. The*recovery rates*of ihMTR and T_1w_-MPRAGE are the only variables loading on PC2 (ρ > 0.7,Fig. 6aandTable 3). Also, the quasi-orthogonality between the vectors of the*recovery rate*variables and the vectors of the*initial loss*and*asymptotic recovery*variables indicates that the recovery rates of ihMTR and T_1w_-MPRAGE in active lesions are not correlated with either their initial loss or their final recovery value. The colored score plots (Fig. 6b-d) help identifying lesions with similar profiles of remyelination based on the additional categorical variables: the overlapping confidence ellipses inFigure 6bindicate that no individual patient profile can be determined based on the investigated active variables. In contrast, the confidence ellipses separated along PC2 (Fig. 6c) suggest a different ihMTR and T_1w_-MPRAGE*recovery rate*profile between proximal CSF and distal CSF lesion groups. The group of lesions distal from the ventricles appears to be associated with faster ihMTR and T_1w_-MPRAGE recovery rates than the group of proximal lesions*.*Quantitative values indicate recovery rates increased 4-fold for ihMTR (2.80 month^-1^vs. 0.63 month^-1^,*p < *0.05) and 2.5-fold for T_1w_-MPRAGE (1.35 month^-1^vs. 0.54 month^-1^*, p > *0.05) for lesions more distal to ventricles (Table 4). Similarly, the confidence ellipses separated along PC1 (Fig. 6d) suggest a different*onset loss and asymptotic recovery*profile between groups of lesions of different sizes. The group of lesions smaller than 200 mm^3^appears to be associated with lower*onset loss*and higher*asymptotic recovery*values of the MR metrics than the group of lesions larger than 200 mm^3^, and vice versa. Specifically, quantitative values indicate an*onset loss*1.2, 1.6, 1.8, and 2.1 times lower for T_1W_-MPRAGE, ihMTR, MTR, and RD, respectively, and an*asymptotic recovery*1.05 and 1.15 times higher for MTR and ihMTR, respectively, for the group of lesions smaller than 200 mm^3^compared with those larger than 200 mm^3^(*p < *0.05, except for T_1w_-MPRAGE,Table 4). Correlation plots between lesion localization and ihMTR/T_1w_-MPRAGE recovery rates, as well as correlation plots between lesion size and onset loss/asymptotic recovery of the relevant parameters extracted from the PCA are provided as Supplementary Material (Fig. S4).

**Table 3.** Summary of the PCA.

		PC1 (44.53%)			PC2 (15.53%)			PC3 (10.53%)		
		Correlation (ρ)	Contribution	cos ^2^	Correlation (ρ)	Contribution	cos ^2^	Correlation (ρ)	Contribution	cos ^2^
IhMTR	Onset loss	**0.94**	16.56	0.89	-0.05	0.16	0.00	0.15	1.83	0.02
	Asymptotic recovery	**-0.80**	11.82	0.63	0.07	0.25	0.00	0.22	3.755	0.05
	Recovery rate	-0.25	1.21	0.07	**0.89**	42.73	0.80	0.09	0.58	0.01
MTR	Onset loss	**0.90**	15.28	0.82	-0.06	0.17	0.00	0.33	8.84	0.11
	Asymptotic recovery	**-0.82**	12.56	0.67	0.15	1.15	0.02	0.35	9.61	0.12
	Recovery rate	0.25	1.14	0.06	0.41	8.90	0.17	-0.19	2.76	0.04
RD	Onset loss	**0.79**	11.77	0.63	0.05	0.12	0.00	0.49	18.64	0.24
	Asymptotic recovery	-0.62	7.22	0.39	-0.15	1.24	0.02	0.21	3.52	0.04
	Recovery rate	0.34	2.12	0.11	0.25	3.30	0.06	-0.48	18.16	0.23
T _1W_ -MPRAGE	Onset loss	**0.82**	12.71	0.68	0.24	3.19	0.06	0.38	11.55	0.15
	Asymptotic recovery	-0.63	7.33	0.39	-0.14	1.07	0.02	0.51	20.24	0.26
	Recovery rate	-0.12	0.28	0.02	**0.84**	37.73	0.70	0.08	0.52	0.01

For each MR parameter, the correlation (ρ) between the first three components and the active variables, as well as their contributions and quality of representation (cos²) are reported. Absolute correlations (|ρ|) above 0.7 (usual criterion for the goodness of representation of a variable onto a specific component) are indicated in bold.

**Table 4.** Quantitative values of the active variables of PCA in lesions.

		Categorical variable separating lesions along PC1	
Active variables loading on PC1		Lesion size < 200 mm ^3^	Lesion size > 200 mm ^3^
Onset loss(NAWM: 0%)	ihMTR*	27.3 ± 9.6%	45.3 ± 14.0%
	MTR*	17.9 ± 5.7%	31.4 ± 12.6%
	RD*	21.4 ± 13.5%	45.1 ± 35.9%
	T _1W_ -MPRAGE	22.9 ± 4.8%	27.9 ± 10.2%
Asymptotic recovery(NAWM:100%)	ihMTR*	89.3 ± 10.7%	77.9 ± 13.4%
	MTR*	91.4 ± 5.6%	87.7 ± 6.2%

		Categorical variable separating lesions along PC2	
Active variables loading on PC2		Lesion > 10 mm from ventricles	Lesion < 10 mm from ventricles
Recovery rate	ihMTR*	2.75 ± 4.72 month ^-1^	0.63 ± 0.59 month ^-1^
	T _1W_ -MPRAGE	1.32 ± 2.42 month ^-1^	0.54 ± 0.46 month ^-1^

Mean values of the active variables loading on the 2 first components (ρ > 0.7) — onset loss of all MR techniques and asymptotic recovery of ihMTR and MTR for PC1; ihMTR and T_1W_-MPRAGE recovery rates for PC2 — calculated in lesion groups with different values of categorical variables, distinct along the 2 first components (lesion size for PC1 and lesion localization for PC2). The symbol * indicates a significant difference between mean values of the active variables calculated in the categorical groups (two-sample*t*-test, p < 0.05, corrected for multiple comparisons — N = 2 for the localization category, N = 6 for the size category).

## 4 Discussion

### 4.1 Changes of metrics in new active MS lesions

Active lesions show Gd+ enhancement, which corresponds with active inflammation in a zone of myelin alteration (Katz et al., 1993). MTR decreases in acute demyelination (Dousset et al., 1992) and increases with remyelination (Deloire-Grassin et al., 2000). In studies on postmortem brain tissues of patients with MS, a strong association between MTR values and myelin content in lesions has been demonstrated (Schmierer et al., 2004). Hence generally, at the time of Gd+ enhancement, a decrease of MTR is observed in MS lesions (Brown et al., 2014;Chen et al., 2008;Giacomini et al., 2009;Lai et al., 1997;Silver et al., 1998). DTI metrics also show changes with WM alterations in MS (Filippi et al., 2001) and sensitivity to demyelination, illustrated by the increase in RD (Song et al., 2005). Hence, despite their lack of specificity for myelin (Oh et al., 2019), these metrics are considered practical markers of demyelination. Thus, the significant decrease in MTR and increase in RD in active demyelinating lesions at t_0_align with these observations. Moreover, the even larger decrease in ihMTR emphasizes its strong association with demyelination processes.

Semiquantitative MTR has limitations in characterizing demyelination due to factors such as edema and inflammatory cell infiltration, which increase the free water volume, thus leading to dilution effects and potential misestimation of myelin density in acute lesions (Fernando et al., 2005;Giacomini et al., 2009). Gliosis, another prominent feature of MS lesions (Moll et al., 2011), also impacts MT and DTI-derived metrics as the dense structure of gliotic tissue would most likely contribute to increased MTR and decreased RD. An interpretation of MTR and RD variations from the perspective of myelination alone would thus overestimate myelin density in lesions. Thanks to T_1D_-filtering, which reduces the contribution of short T_1D_components (of which gliosis would be a part (Hertanu et al., 2022)) to the total ihMT signal, a lesser impact of gliosis is expected on ihMTR values. More generally, the greater specificity of ihMT for myelin may explain its stronger variations in active lesions compared with other metrics (Fig. 3). At t_0_, RD and MTR relative variations align with ihMTR, potentially indicating that demyelination processes drive MR metrics changes during Gd+ enhancement. However, at M_12_, the variations in ihMTR (relative to NAWM) were 1.6 times and 1.8 times higher than that of MTR and RD, respectively, which suggests that nonmyelin-related processes contribute to MTR and RD. This hypothesis is further supported by the intra-parameter correlations. The strong association for ihMTR between t_0_and M_12_suggests a physiological progression related to increased myelination. Lack of correlation for MTR and DTI between the two time points implies that additional processes beyond remyelination influence signal variations. In other words, and without commenting on any underlying pathophysiological mechanisms, the stronger intraparameter correlations between t_0_and M_12_suggest that the onset loss of signal is more predictive of the final recovery value for ihMT than for any other tested MR techniques.

### 4.2 Dynamic model of MR metrics recovery in active lesions

MT-based imaging stands out as the most utilized advanced MRI technique in MS research studies, and longitudinal changes of MTR in various lesion types have been widely reported (York et al., 2022). The dynamics of MTR during the lesion evolution is heterogeneous and depends on lesion type as synthesized in thesupplementary Figure 3inYork et al. (2022). However, active lesions typically display a drop in MTR at the time of Gd enhancement followed by a 5- to 6-month period of partial recovery—indicative of demyelination followed by partial remyelination (Brown et al., 2013,^2014^;Chen et al., 2008;Filippi et al., 1998,^1999^;Giacomini et al., 2009;Goodkin et al., 1998;Lai et al., 1997;van Waesberghe et al., 1998). This MTR recovery profile in active lesions aligns with the canonical form shown in Figure 2 fromOh et al. (2019)and can reasonably be modeled by an exponential recovery model, as employed in our study.

Our analyses only focused on the core of active lesions, identified by the T_1w_-hypointense area at baseline, assumed to exhibit the highest WM alteration and myelin disruption. The lesion edges, representing the area between the entire lesion and its core and showing a higher T_1w_signal at baseline, were not considered. This decision was motivated by the signal dynamics observed in this area. For a significant number of lesions, the signal at the edges returned to almost isointense with the NAWM rapidly (within the first 2 months, e.g.,Fig. 2) and was more indicative of a dominant inflammation or edema at baseline that rapidly washes out, rather than demyelination–remyelination processes. In addition, the width of the peripheral zone for some lesions was not always as large as that shown inFigure 2. In these cases, we suspected significant partial volume effects with the outer NAWM but also with the lesion core. Overall, we hypothesize that the signal dynamics at the edges of lesions is due to a complex mix of pathological mechanisms including demyelination–remyelination and inflammation–resorption and partial volume effects, making its interpretation extremely difficult. We provided in the Supplementary Material some representative examples of the signal observed in the core and edge of lesions, highlighting different temporal dynamics (Fig. S5). In the lesion edges, the exponential recovery model failed for more than 75% of the lesions for ihMTR and more than 50% for the other metrics. Conversely, in the lesion cores, the model successfully fitted the evolution profile of MR metrics (R²_adj_> 0.6) for ~80% of the studied active lesions. This figure is similar to that of an immunohistochemical study carried out on biopsies from MS patients in search of signs of remyelination in early lesions (Goldschmidt et al., 2009). While partial volume effects and low SNR, particularly in RD and ihMTR, may have contributed to the fit failure in the remaining 20% of lesions, some lesions displayed ihMTR/MTR profiles without recovery or even with decay, reflecting the diverse evolution patterns reported previously (Filippi et al., 1999;van Waesberghe et al., 1998). This variability is linked to heterogeneity in longitudinal signal changes from individual voxels in a lesion induced by opposing processes (e.g., degeneration vs. repair) and hidden in the mean value over all lesion voxels (Chen et al., 2008;Meier & Guttmann, 2006). Including a degenerative component in addition to the recovery component for a more comprehensive model (Meier & Guttmann, 2006) would allow for a more textural characterization of lesions, but at the cost of more MRI examinations. The exponential recovery behavior of MRI metrics, and in particular MTR, observed here in active lesions may somewhat align with a previous study examining the behavior of prelesion changes in MTR and describing an exponential decrease in MTR prior to lesion onset (Laule et al., 2003). Hence, a more extensive longitudinal follow-up using multimodal MR, covering both the prelesional phase and the recovery phase, could perhaps allow a more detailed characterization of certain mechanisms.

### 4.3 Features of remyelination in active MS lesions

The simple model used enabled detailed characterization of active MS lesions recovery. PCA incorporating model parameters from the four myelin-sensitive techniques identified distinct recovery profiles based on lesion size and localization. Normalized signal variations (% of signal change in lesions compared with contralateral NAWM) enabled common units for all MRI metrics and thus consistent interpretation of the PCA variables. Hence, the significant contribution of ihMT-related variables to principal components (>29% for*onset loss*and*asymptotic recovery*to PC1 and >42% for*recovery rate*to PC2, ρ > 0.7), coupled with ihMT’s proven myelin specificity (Duhamel et al., 2019;Hertanu et al., 2022), provides further support that the identified recovery profiles are myelin related and not linked to any other physiological process. PCA results also suggest that ihMT, in combination with the less specific T_1w_-MPRAGE (whose variables contributed >20% to PC1 and >39% to PC2) typically acquired anyway, could be an excellent candidate for a remyelination marker in therapeutic studies.

The dynamics of ihMT as modeled in our study can be mainly interpreted from the perspective of demyelination and remyelination and the PCA results may associate with some previously reported results. It is indeed now well acknowledged that a periventricular gradient of microstructural damage in both NAWM and demyelinating lesions characterizes early and established MS. Lower MTR (Brown et al., 2017;Liu et al., 2015;Pirpamer et al., 2022), longer T_1_(Kolb et al., 2021;Vaneckova et al., 2022), and higher [^18^F ]-based PET (Poirion et al., 2021) signal were found in periventricular lesions compared with subcortical ones. In terms of evolution, immunohistochemistry analyses have shown that subcortical and deep white matter lesions present more extensive remyelination than periventricular lesions. These observations were confirmed by quantitative T_1_analyses where subcortical lesions were more likely to evolve into short-T_1_lesions (suggestive of remyelination), whereas juxtacortical and periventricular lesions were more likely to become long-T_1_lesions (suggestive of demyelination) (Kolb et al., 2021). The reasons for such a difference and whether the mechanisms for remyelination (OPC proliferation, recruitment, differentiation, and maturation (Franklin & ffrench-Constant, 2008)) are location-dependent are not yet elucidated. Our study did not address this issue, but the more than 4-fold higher ihMTR recovery rate (2-fold for T_1w_-MPRAGE), suggestive of a 4-fold higher remyelination speed (2-fold for MPRAGE) in lesions distant from the ventricles than in proximal lesions, confirms that subcortical lesions are more conducive to remyelination than periventricular lesions. Nevertheless, an important nuance to the previous location-related findings must be noted. According to the PCA, the extent of remyelination, which can be assessed by the*ihMTR*(and other MR metrics)*asymptotic recovery*, does not correlate with the lesion localization, but negatively correlates with the lesion size, suggesting that remyelination extent may be impaired in large lesions despite being distant from ventricles. In other words, small subcortical lesions will remyelinate faster and to a higher level than small periventricular lesions, and large subcortical lesions will remyelinate faster than large periventricular lesions, but not necessarily reach a higher level. More generally, the size effect observed here and characterized by the association of small lesions with low extent of demyelination (small ihMTR and other MR metric*onset losses*) and high extent of remyelination (high ihMTR and other MR metric*asymptotic recoveries*)—the opposite applying for large lesions—supports previous quantitative T_1_studies, which report that small lesions were more likely to evolve into short-T_1_lesions (Kolb et al., 2021) compared with large ones. It should be mentioned that the genuine onset loss should have been measured at time of lesion appearance. Here instead, the onset loss used in the exponential recovery model was calculated based on the parameters’ value at the first time point at which an active lesion could be evidenced by MRI (i.e., M_0_or M_2_), and assuming that gadolinium activity persisted for less than 4 weeks. This assumption is reinforced by a study in which weekly follow-up was carried out on RRMS patients, which showed that over 50% of new active lesions had an enhancement duration of less than 3 weeks, and almost 80% a duration of less than 4 weeks (Cotton et al., 2003). However, this study also found that enhancement duration was positively correlated with lesion size and that lesions greater than 400 mm^3^may show enhancement during more than 8 weeks. In consequence, large lesions detected at M_0_in this study might be older than 4 weeks, and hence the associated onset loss value might have been significantly underestimated, thus potentially confounding the differences observed as a function of lesion size. Note, however, that this should have limited impact on the estimation of the asymptotic recovery values and recovery rates (beyond noise and uncertainties), thanks to the mathematical properties of the exponential recovery model. One way of avoiding the potential effect of underestimated onset loss values associated with older lesions would be to perform the analysis only on new lesions detected at M_2_(provided they are sufficiently numerous), which by definition would have been active for less than 8 weeks.

Nonetheless, the size effect observed here is also consistent with a series of observations that OPC migration from the area surrounding the lesion to areas of demyelination occurs only over a very short distance (~1 mm) during repair (Franklin & Blakemore, 1997), thus limiting the remyelination capabilities of large lesions. Of note, a work modeling the dynamics of T_2w_hyperintensities in lesions with two opposite processes of longitudinal intensity change, such as inflammation and degeneration versus resorption and repair (Meier & Guttmann, 2006;Meier et al., 2007), showed that after 12 weeks, the proportion of residual T_2_hyperintensity (suggestive of damage) is substantially smaller in larger lesions compared with small ones. This seems to contradict our results, however, given the lack of specificity of T_2w_contrast and its sensitivity to multiple different mechanisms, it is possible that this observation is linked to the effects of reduced inflammation or edema resorption, rather than to remyelination mechanisms.

There is evidence for a high interpatient variability of myelin content change and that the extent of remyelination in MS lesions in response to a demyelinating insult is patient dependent. According to the PCA results, no patient effect was observed, suggesting that none of the variables in the dynamic model studied is relevant for determining an individual remyelination profile. However, given the small number of patients in this study, this result needs to be confirmed.

### 4.4 Stability of MR metrics in normal WM

Long-term stability in metrics from t_0_to M_12_in NWM, with low CoVs (<1%), suggests insensitivity to physiological changes over time. Consequently, MR metric variations beyond these CoV values might indicate alterations induced by pathological processes in patients.

### 4.5 Changes of metrics in contralateral NAWM

Histopathological evidence in MS indicates that pathological abnormalities in NAWM primarily involve axonal damage and loss, as well as intense microglial activation, whereas no major demyelination seems involved (Kutzelnigg et al., 2005;Moll et al., 2011). This is further confirmed by the normal range of the binding values of the [^11^C]PiB myelin-sensitive PET tracer obtained in NAWM of patients with MS (Bodini et al., 2016). Furthermore, Moll et al.’s study (Moll et al., 2011) combining*in situ*postmortem multimodal MRI and histopathology demonstrated that axonal degeneration and microglial activation accordingly accounted for the decrease of MTR and the increase of RD in NAWM tissues. Therefore, the subtle significant changes in metrics observed here in NAWM versus NWM, consistent with previous literature that reported a slight decrease in MTR (Liu et al., 2015) and an increase in RD (Kim et al., 2017) in NAWM of RRMS patients, likely result from nonmyelination-related tissue alterations. Nonetheless, it is unclear whether they reflect a direct sensitivity to these tissue changes, or whether they are due to a decrease in myelin density in the imaging voxels because of cellular entry accompanying tissue alterations. However, in accordance with other longitudinal studies using MTR (Filippi et al., 1998;Rocca et al., 1999;Rovira et al., 1999), the MR metrics’ values measured in NAWM in this study remained stable over time, which supports using contralateral NAWM as a reference for calculation of the relative variations of metrics within the core of lesions.

### 4.6 Limitations

The small patient cohort reduces the reliability of individual analyses (patient effect), warranting exploration in a larger cohort. In addition, a better match between the control group and the patients in terms of sex and age than in this study would also be warranted to avoid potential biases. Analyses on all lesions, however, were sufficiently large to reveal effects of lesion size and localization on remyelination. Correlation analyses (Fig. S4) underlined that a larger number of lesions would help in increasing these associations. The ihMT technique’s relatively low SNR at 1.5T limited spatial resolution to 2 mm, with potential partial volume effects. The lower SNR of ihMTR also explains the higher number of discarded lesions because of poorer fits compared with MTR. Transitioning to 3T with ihMT saturation schemes immune to transmit field B_1_^+^inhomogeneities (Munsch et al., 2021;Soustelle et al., 2022) can overcome this, allowing higher resolution (~1.5-mm isotropic).

Measures derived from MT and T_1w_techniques were semiquantitative, and longer T_1_values could partially offset ihMTR and MTR changes. More quantitative ihMT and MT approaches may enhance sensitivity for characterizing MS lesion recovery.

## 5 Conclusion

This work focused on characterizing the recovery of active MS lesions by modeling the lesion signal dynamics of myelin-sensitive MR metrics of variable specificity, including ihMTR, MTR, RD, and T_1w_-signal. A time-recovery exponential model was applied on the signal variations over a 12-month follow-up period for all metrics. Combining the model parameters from the four myelin-sensitive techniques in a principal component analysis enabled us to identify specific recovery profiles according to lesion size or localization. An association was established between lesion location relative to the ventricles and the ihMT and T_1w_-MPRAGE signal recovery rate. An association was also established between lesion size and the initial loss as well as final recovery of the ihMT and other MR techniques signal. Thanks to the specificity of ihMT for myelin, these features can be interpreted in terms of remyelination, and are in line with literature data based on*ex vivo*analyses using histological myelin markers. This leads to the conclusion that the ihMT technique has a promising potential as an*in vivo*marker of remyelination that could be used to monitor patients and evaluate the efficacy of various therapies targeting myelin recovery.

## Supplementary Material

Supplementary Material

## Data Availability

Data will be shared upon request from any qualified investigator. The ihMT preprocessing pipeline (including denoising, Gibbs-ringing artifact correction and motion correction) is available at:https://github.com/lsoustelle/ihmt_proc(hash #c9bb409).
