# Supplementary material for: Inhomogeneous magnetization transfer (ihMT) imaging reveals variable recovery profiles of active MS lesions according to size and localization
Source: Imaging Neurosci (Camb). 2024 Jul 24;2:imag-2-00235. doi: 10.1162/imag_a_00235 (PMC12272197; doi:10.1162/imag_a_00235)
Supplement: Supplementary Material [file imag_a_00235-supp.pdf]

Inhomogeneous Magnetization Transfer (ihMT) imaging reveals variable recovery profiles of active MS lesions according to size and localization

Lucas Soustelle[1,2], Samira Mchinda[1,2], Andreea Hertanu[1,2], Soraya Gherib[1,2], Lauriane Pini[1,2], Maxime Guye[1,2], Jean-Philippe Ranjeva[1,2], Gopal Varma[3], David C. Alsop[3], Jean Pelletier[1,2,4], Olivier M. Girard[1,2], Guillaume Duhamel[1,2,*]

1. **Aix Marseille Univ, CNRS, CRMBM, Marseille, France**
2. **APHM, Hôpital Universitaire Timone, CEMEREM, Marseille, France**
3. **Division of MR Research, Radiology, Beth Israel Deaconess Medical Center, Harvard Medical School, Boston, MA, United States**
4. **APHM, Hôpital Universitaire Timone, Service de neurologie, Marseille, France**

* Corresponding author: G. Duhamel (guillaume.duhamel@univ-amu.fr), Aix Marseille Univ, CNRS, CRMBM UMR 7339, 27 bd Jean Moulin, Faculté de Médecine, 13385, Marseille, France

Supplementary Material

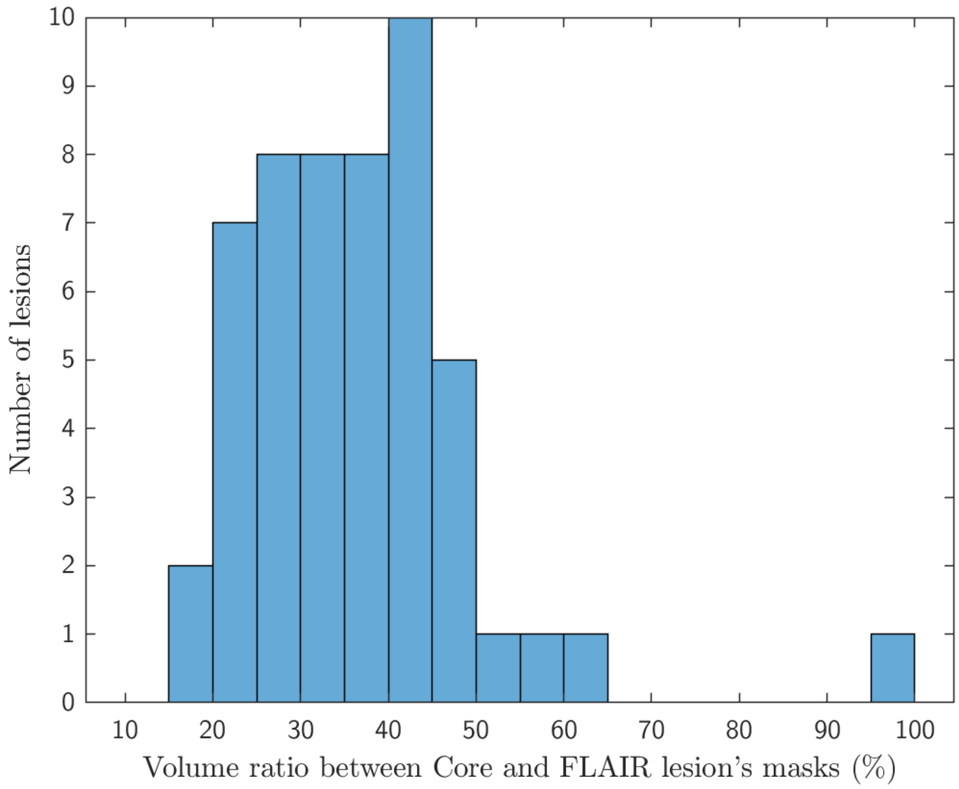

Figure S1: Distribution of ratios between the core volume and the entire volume of active lesions.

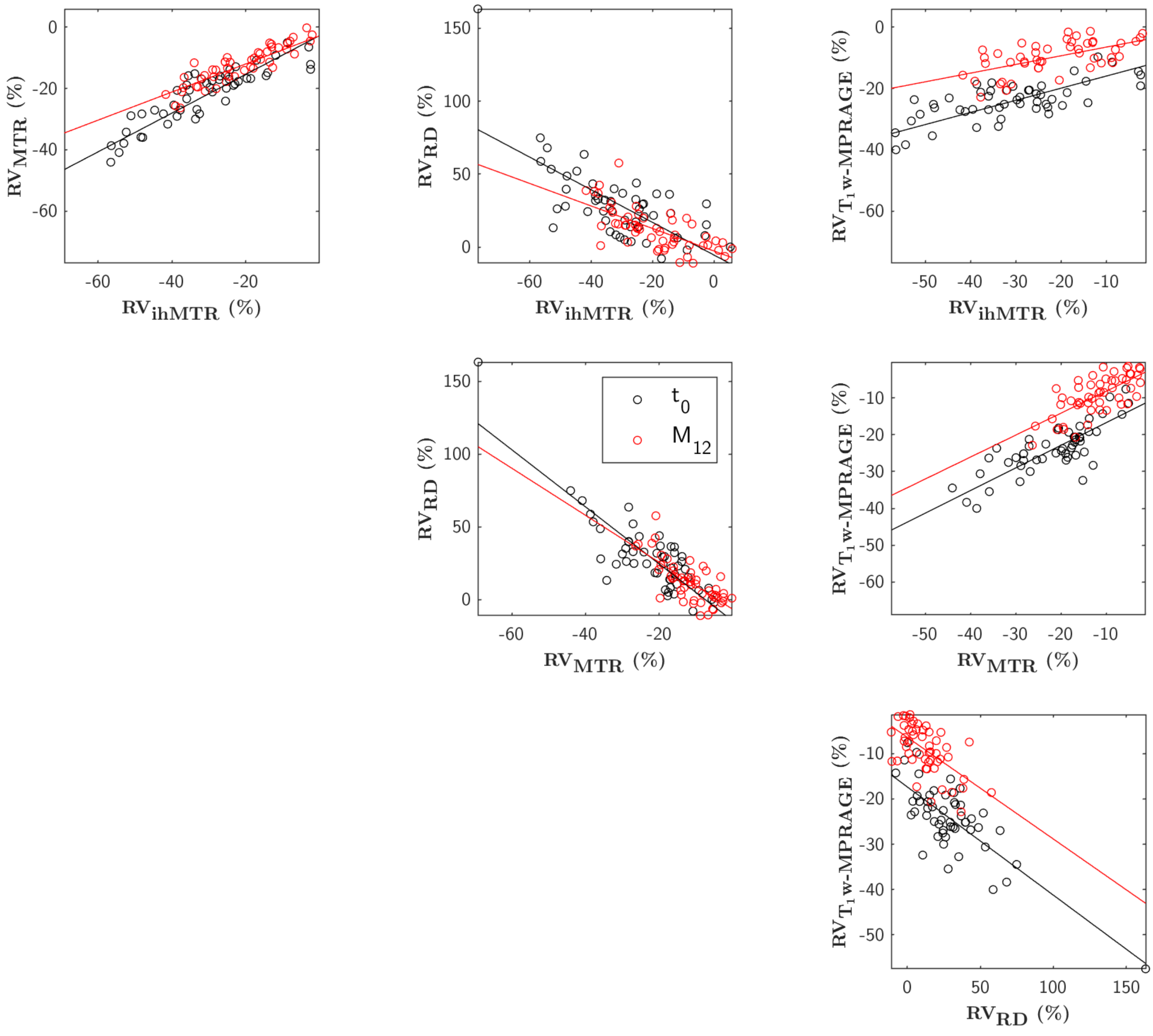

Figure S2: Inter-correlation plots of MR metrics at t_0 (black) and M_{12} (red).

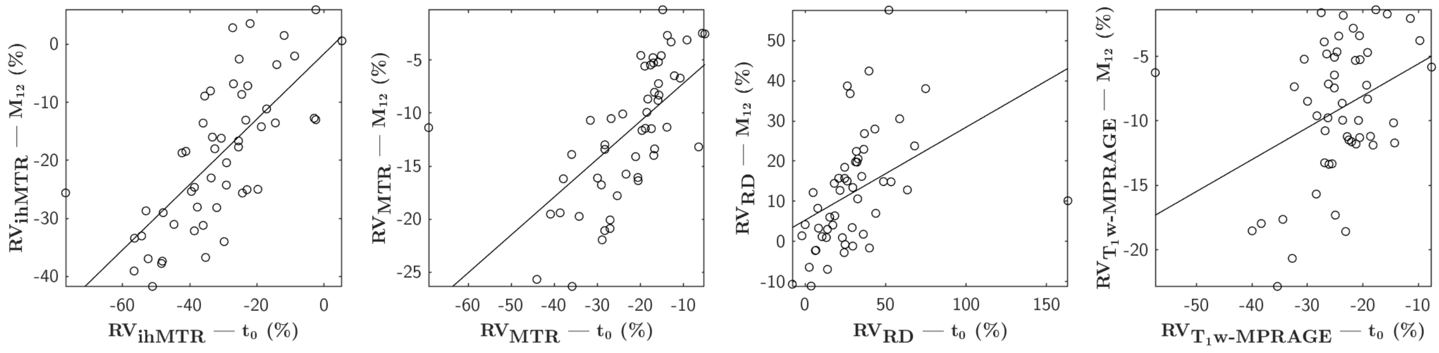

Figure S3: Intra-correlation plots of MR metrics between t_0 and M_{12}.

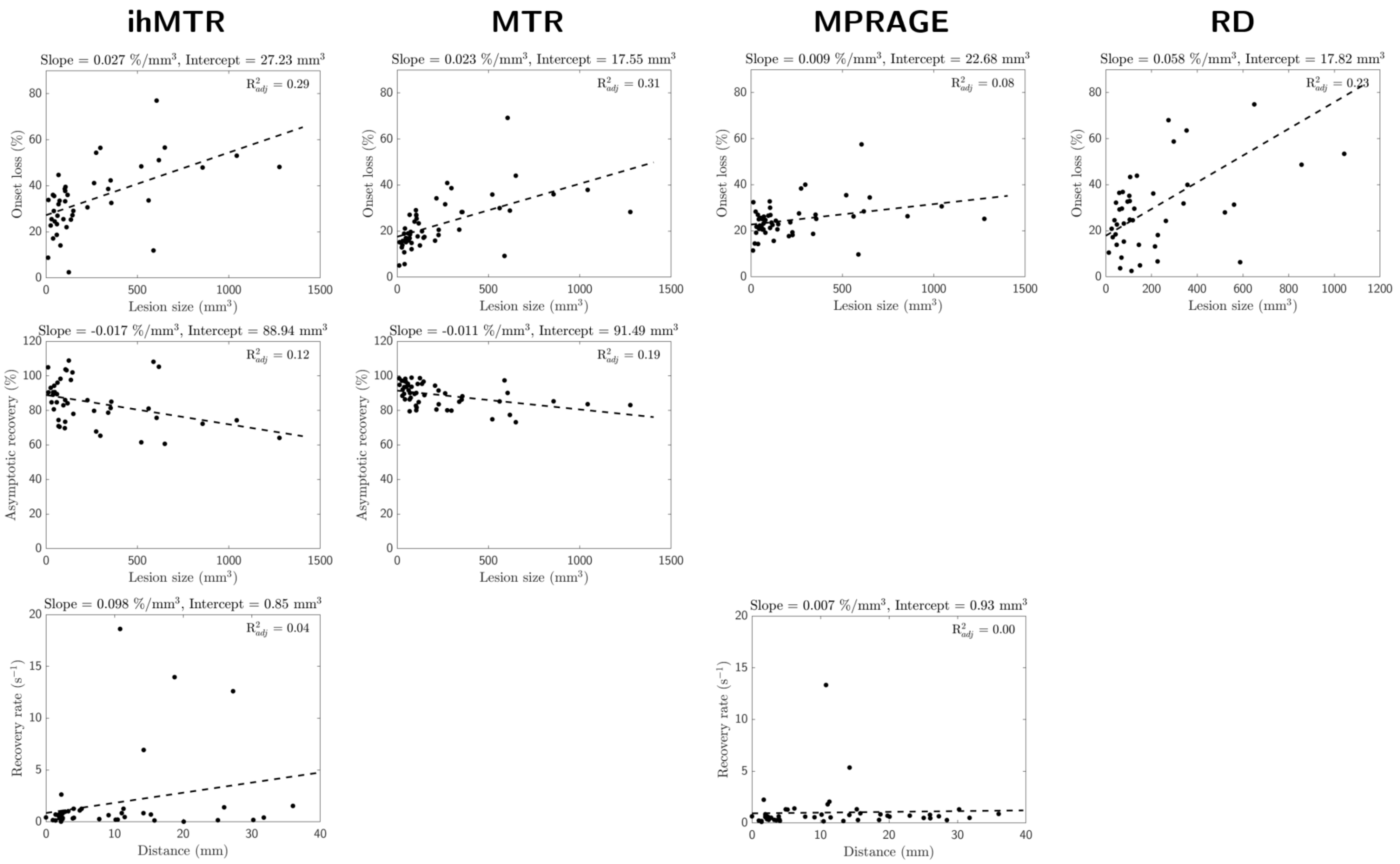

Figure S4: Correlations of ihMTR, MTR, T_{1w}-MPRAGE and RD derived metrics (onset loss, asymptotic recovery and recovery rate) against the relevant lesions' features (distances to lateral ventricles and sizes at baseline) as identified by the principal component analysis.

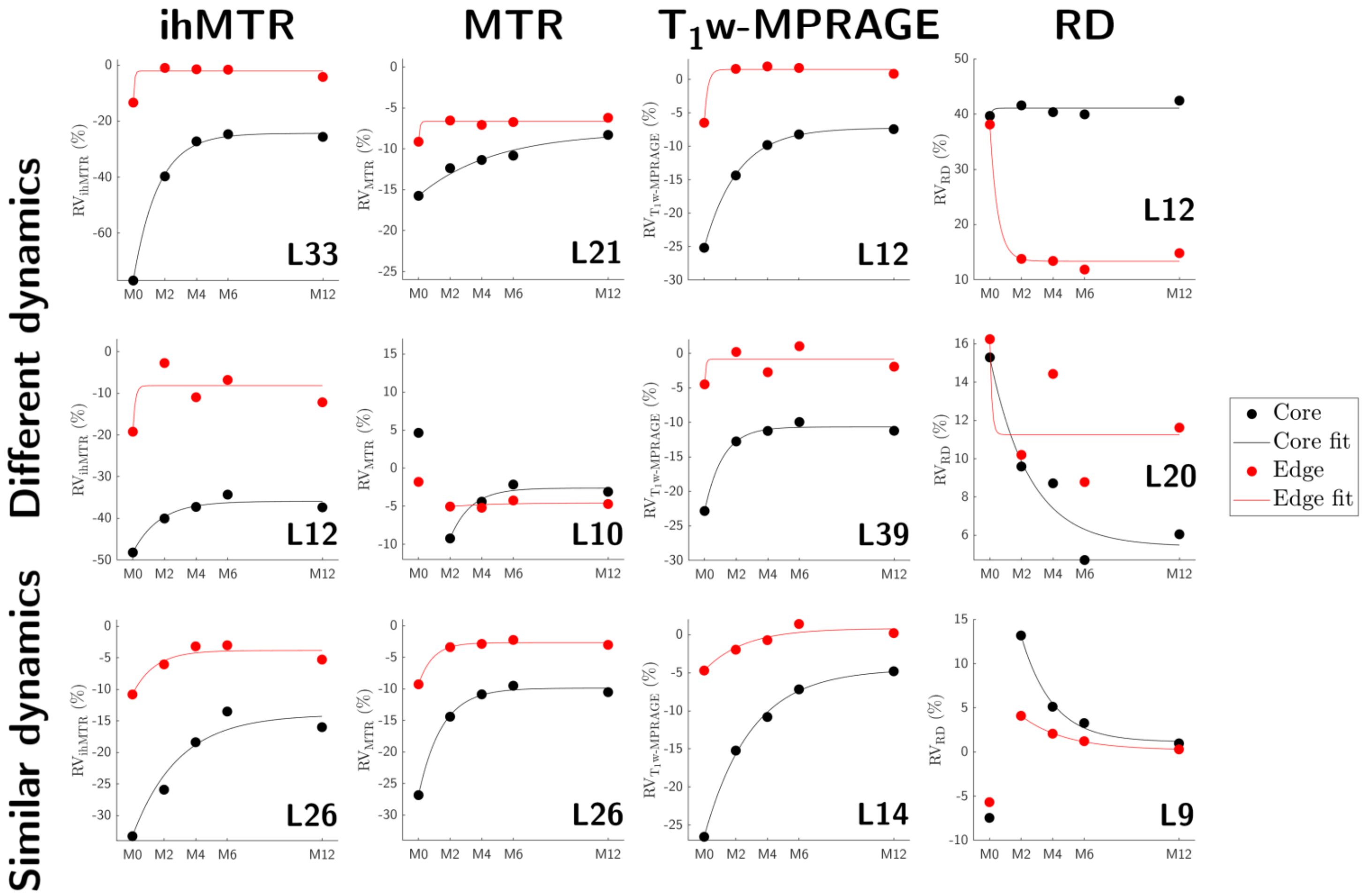

Figure S5: Temporal dynamics of MR metrics observed in the cores (black) and edges (red) of different lesions. Top and middle rows highlight different dynamics between core and edge. Top row: rapid return (within 2 months) of MR metric signals close to NAWM isosignal in the edges; exponential recovery for ihMTR, MTR and T_{1w}-MPRAGE and stability for RD in the cores. Middle row: no clear dynamics (neither recovery nor reduction) at the edges; exponential recovery in the cores. Bottom row: similar exponential recovery dynamics of MR metrics in both the edges and the cores.
